# Measuring corrosion rate and protector effectiveness of advanced multilayer metallic materials by newly developed methods

**DOI:** 10.1016/j.heliyon.2018.e00731

**Published:** 2018-08-14

**Authors:** Vladimir A. Grachev, Andrey E. Rozen, Yuri P. Perelygin, Sergey Yu. Kireev, Irina S. Los', Andrey A. Rozen

**Affiliations:** a A.N. Frumkin Institute of Physical Chemistry and Electrochemistry, Russian Academy of Sciences, 31, Bldg 4, Leninsky Prospect, Moscow, 119071, Russia; b Penza State University, 40, Krasnaya St., Penza, 440026, Russia

**Keywords:** Materials science, Electrochemistry

## Abstract

The paper estimates corrosion resistance of new multilayer metallic materials with internal protector against pitting. Using an electron microscope method, the mechanism of the layers' corrosive destruction has been experimentally substantiated. The authors have suggested chemical and electrochemical methods of accelerated corrosion tests allowing for determining the corrosion destruction rate. The electrochemical method reveals the limiting stage of the process and allows calculating the mass corrosion index and substantiating the choice of protector for the specific corrosive medium. The chemical method allows for quantitative assessment of the internal protector's effectiveness and for defining the multilayer/monometallic material corrosion resistance ratio.

## 1 Introduction

The issue of pitting corrosion has been a challenge for decades in the context of wide application of high alloy steels and alloys in different segments of industry. The main research trends in this field are the development of new materials and testing methods, studies of the industrial materials' behavior in various mediums, and simulation of pitting formation.

Creation of a new class of multilayer metallic materials with internal protector can be the solution to the problem of protection against pitting [1, 2]. Such materials contain layers that have different electrochemical potentials and form short-circuited galvanic elements. This architecture fundamentally changes the process of corrosion and extends the material's operating life. The kinetics of the multilayer metallic material's corrosive destruction and the mechanism of the internal protector's action require further study.

Application of new materials is impossible without the development of reliable methods of accelerated corrosion tests, adapted to the given architecture.

The existing methods of accelerated corrosion tests determining the resistance against pitting [3, 4, 5] estimate the corrosion rate of monometallic materials. According to GOST 9.912-89 and ASTM G48-11, the stability of steels and alloys is evaluated using the chemical method, which consists in determining the mass corrosion index. A sample is placed in a thermostatically controlled vessel with 6% FeCl_3_·6H_2_O solution for a specified time. After the soaking and removing the corrosion products, the average conditional rate of pitting corrosion in terms of mass loss is weighed and calculated. According to GOST 9.912-89, the electrochemical tests imply determination of the pitting resistance when measuring the free corrosion potential. The basic and additional bases of pitting resistance are calculated.

In addition to the standard test methods, private methods have been developed and patented. To identify the propensity of steels and non-ferrous metals to pitting corrosion, they use electrochemical methods that differ in the electrical circuit, composition of the electrolyte, and number of electrodes [6, 7, 8]. In [9], a method for the computerized image processing of the investigated surface in the initial state and after exposure to the medium with a subsequent analysis of the pitting growth dynamics was developed for the purpose of modeling and forecasting. The measured index is the perimeter, along which the equivalent diameter is determined, and then, with a known wall thickness, the time of the item's safe operation is calculated. In [10, 11], the methods based on the surface 3D metrology for studying and predicting the pitting corrosion were proposed to determine the rate of the pitting increase in linear dimensions and depth.

The [12] considers a method that made it possible to reveal the presence of a limited zone of the internal protector's protective action. At the same time, it does not allow evaluation of the kinetic regularities and quantitative estimation of the corrosion rate for each layer separately by the gravimetric method.

The authors of the article propose the methods of accelerated corrosion tests of multilayer metallic materials, designed to calculate quantitative indicators.

The purpose of this work is to study the specific features of the corrosion mechanism in a new class of multilayer metallic materials with internal protector and to develop methods of accelerated corrosion tests for them.

## 2 Theory

**Fig. 1:**
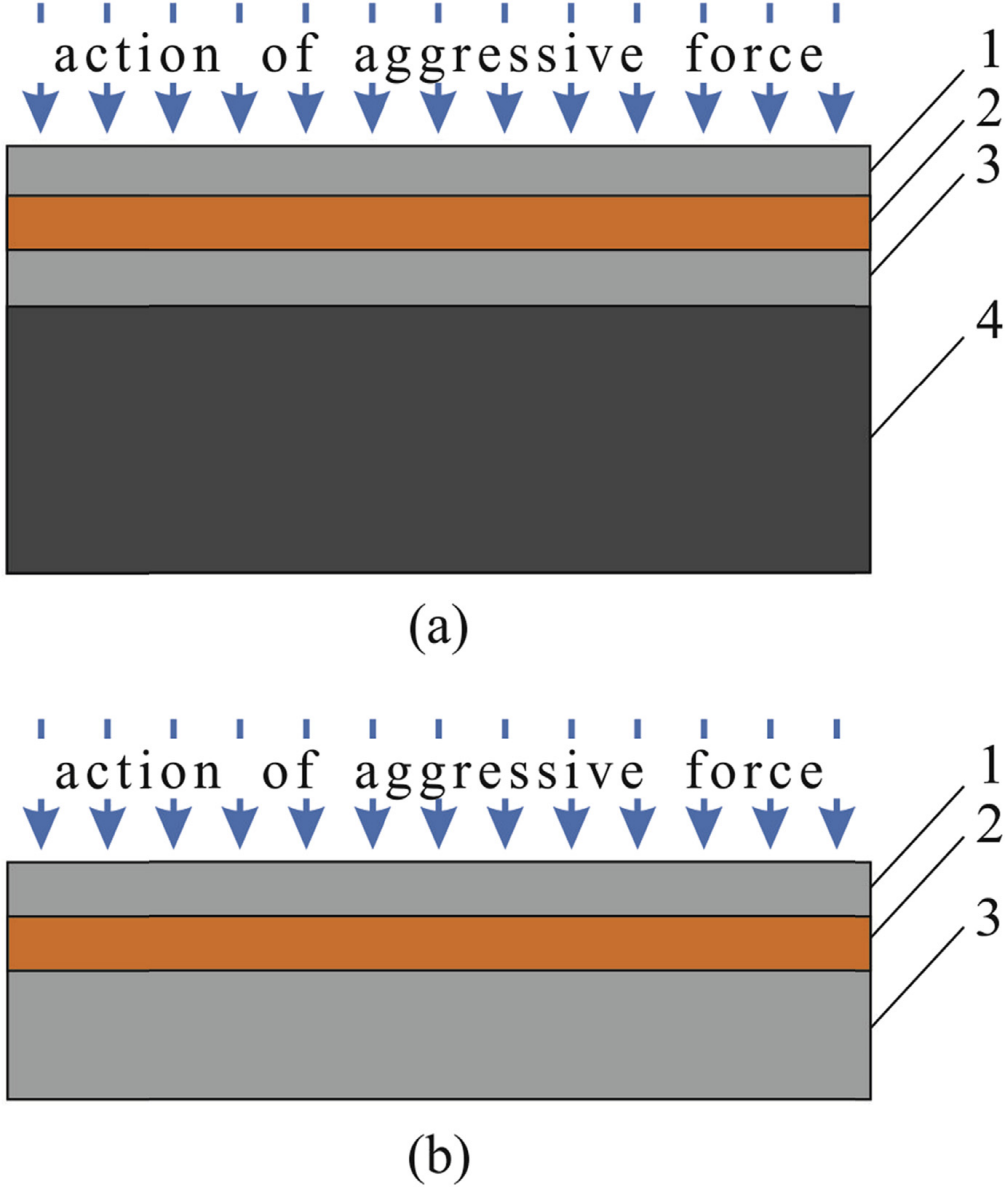
Schematic view of four-layer (a) and three-layer (b) metallic materials with internal protector: 1 – layer with high electrochemical potential, exposed mainly to pitting corrosion; 2 – layer with low electrochemical potential (“sacrificial layer” – internal protector); 3 – layer with high electrochemical potential; 4 – layer providing mechanical strength.

This engineering approach is based on a multilayer material comprised of the components with different electrochemical potentials; these components transform the corrosion processes upon transition from one layer to another. The corrosion damage is transformed from pitting corrosion in the upper layer into contact corrosion of the sacrificial layer. In case of unilateral impact of aggressive medium, four- and three-layer compositions are used. In case of items operating under high internal or external loads and pressures, four-layer compositions are used (Fig. 1a). The first three layers provide corrosion protection of material, and the fourth layer is for mechanical strength. If the material is not exposed to significant loads, three-layer compositions are used. In this case, the thickness of the third layer is determined by the strength calculation (Fig. 1b) [13, 14, 15, 16].

When a multilayer material contacts with corrosive medium, the first layer is destroyed. Under certain conditions, there occurs pit nucleation and growth in the external layer.

When the first pitting reaches the middle layer, a short-circuited galvanic element is formed, in which the second layer material acts as an anode. After that, it is the second layer that is exposed to corrosion damage, providing galvanic protection of the first and third layers. In this case, the pitting growth rate decreases since the pitting surface potential becomes negative. Herewith, on the pitting surface, instead of metal dissolution, there occur reactions determined by the medium composition. In general case: Ox^z+^ + ze = Red; in particular, hydrogen evolution or oxygen reduction: 2H^+^+2e = H_2_ in acid medium, 2H_2_O + O_2_ +4e = 4OH^–^ in neutral and alkaline medium. On the surface of the second protecting layer, metal dissolution starts: M^0^ = M^+z^ + ze. During simultaneous contact of the first, second, and third layer with corrosive medium, the protector's action will extend both to the first and third layers.

The internal protector's functioning mechanism and radius of the protecting action are determined by the effectiveness of the formed galvanic element, namely [17, 18]:

1) the electromotive force (EMF) determined by the difference in the electrochemical potentials of the materials of the first and second layers;
2) the corrosion current that depends on the EMF and composition of corrosive medium inside the pitting.

The composition of corrosive medium inside the pitting can differ significantly from the composition of corrosive medium surrounding the multilayer material.

Generally, the rate of aforementioned reactions can be described as follows [19]:

(1) $$i_{c}=z_{c}Fk_{c}{\left[Ox^{z+}\right]}^{p}\text{exp}\left(-\alpha z_{c}EF/RT\right)\text{,}$$

(2) $$i_{a}=z_{a}Fk_{a}\text{exp}\left(\beta z_{c}EF/RT\right)\text{.}$$

*i*_*c*_ and *i*_*a*_ are the current densities of cathodic and anodic reactions, *z*_*c*_ and *z*_*a*_ are the numbers of electrons participating in cathodic and anodic processes, *F* is the Faraday constant, *k*_*c*_ and *k*_*a*_ are the constants of rate of cathodic and anodic processes, [*Ox*^*z+*^] is the molar concentration of an oxidizer, *p* is the reaction order with respect to the ions of oxidizer, *α* and *β* are the transfer coefficients of cathodic and anodic reactions, respectively, *E* is the compromise potential of the “first layer–second layer” system, *R* is the universal gas constant, *T* is the absolute temperature.

In case of short-circuited galvanic element, the cathodic current (*I*_*с*_) equals to the anodic current (*I*_*a*_):

(3) *I*_*c*_ = *I*_*a*_ or *S*_*a*_*i*_*a*_ = *S*_*c*_*i*_*c*_.

*S*_*a*_ is the surface area of protector, *S*_*c*_ is the cathodic surface area.

Combined solution of Eqs. (1), (2), and (3), with consideration that the potentials of protector and metal of the pitting are equal, results in the following equation:

(4) $$\ln\frac{S_{a}}{S_{c}}=\ln\frac{z_{c}k_{c}}{z_{a}k_{a}}+p\;\ln\left[Ox^{z+}\right]-\frac{FE\left(z_{c}\alpha +z_{a}\beta \right)}{RT}\text{.}$$

From the Eq. (4) it follows that the ratio of the surface areas of protector and cathodic surface depends on the concentration of depolarizer, the ratio of the rate constants of cathodic and anodic reactions and compromise potential *E* which is between stationary potentials of the external layer's material (*E*_*ex*_) and the protector's material (*E*_*pr*_) in the considered solution [17, 18, 19]. The more negative the stationary potential of protector is, the higher the ratio of surface areas of cathodic surface (*S*_*с*_) and protector (*S*_*a*_) is.

Analysis of the Eq. (4) also makes it possible to assume that the radius of the protector's action can be changed by selecting the materials of the first and second layers. And the more the potential of the second layer (internal protector) is negative, the more efficient the protector is.

When choosing a material for the protector, besides electrochemical properties it is necessary to take into account mechanical, technological properties, as well as economic feasibility.

The obtained equation contains parameters that cannot be determined experimentally, but allows explaining the observed regularity of the corrosion behavior of a three-layer material at a qualitative level [17, 18, 19].

The concentration of depolarizer in the pitting pore decreases with time, and the electrolyte conductivity also decreases, thus reducing the area of the protected surface. The pit can be filled with the products of the protector's dissolution; as a result, the action of short-circuited galvanic element ceases. New pitting centers are formed on the external layer's surface.

The compromise potential is determined by the limiting stage running on the cathode or anode [20]. Determination of the corrosion products' composition allows establishing the mechanism and sequence of the corrosion destruction of the layers.

The existing methods of accelerated tests, based on the control of the sample's weight loss and pitting depth [3, 4, 5], do not provide accurate forecast of this integral materials' behavior, which requires for new approaches.

## 3 Experimental

The principle of sacrificial protection against pitting was studied on a three-layer material composed of 08X18H10T (AISI 321 analog) + steel 10 (ASTM 1010 analog) + 08X18H10T, obtained by the explosion welding technology. The composition of steels and their analogs is indicated in Table 1 [21, 22, 23, 24, 25].

**Table 1:** The composition of steels.

Steel grade	Mass fraction of elements, %										
	С	Сr	Ni	Ti	Cu	Si	Mn	S	P	N	Fe
								No more			
08X18Н10Т	0.08 no more	17.00–19.00	9.00–11.00	5·С-0.70	-	0.70 no more	2.00 no more	0.020	0.040	-	Basic
AISI 321	0.08	17.00–19.00	9.00–12.00	5·(С+N)-0.70	-	1.0 no more	2.00 no more	0.030	0.045	-	Basic
Steel 10	0.07–0.14	0.10 no more	0.30 no more	-	0.30 no more	0.17–0.37	0.35–0.65	0.035	0.030	-	Basic
ASTM A29 1010	0.08–0.13	-	-	-	-	-	0.30–0.60	0.04	0.05	-	Basic

Explosion welding is widely used for joining layers of a large area of a wide range of materials [26].

A qualitative assessment of the stated principle's effectiveness was carried out on the samples cut from sheet material with an area of 9.0 m^2^ (sample dimensions were 100 × 200 mm, layers thickness was 2.0 mm). The samples were exposed to a solution of iron (III) chloride (density 1.049 ± 0.002 g/cm^3^) at a temperature of 23 ± 2 °C [3].

**The electron microscope studies** were carried out using a FEI HELIOS NANOLAB 660 double-beam scanning electron microscope equipped with an attachment for the energy-dispersive micro-X-ray spectral analysis EDAX.

**Fig. 2:**
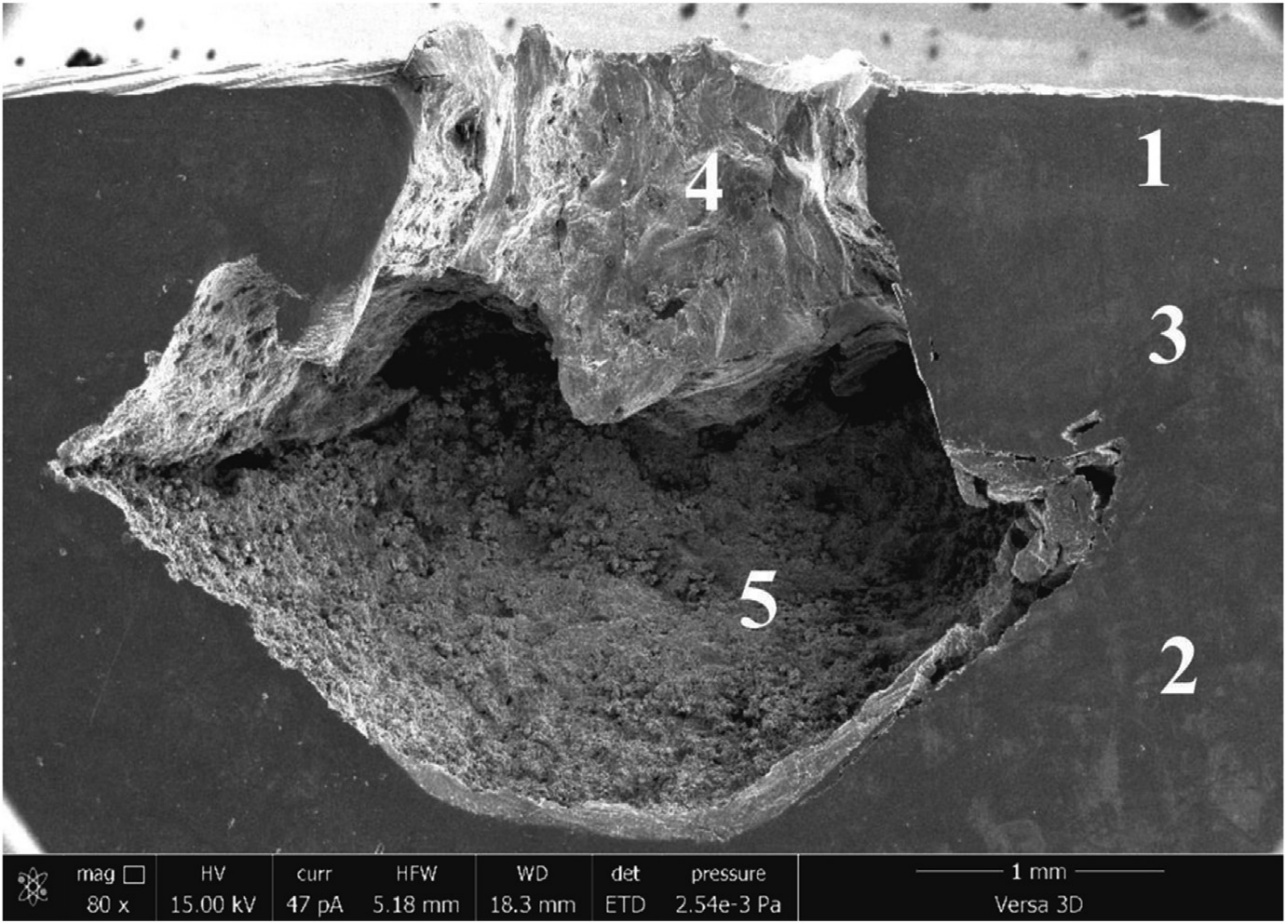
Cross section of natural pitting of a three-layer sample under conditions when the corrosion does not reach the third layer. 1 – external layer, 2 – internal protector, 3 – interlayer boundary, 4 – natural pitting, 5 – lens.

**Fig. 3:**
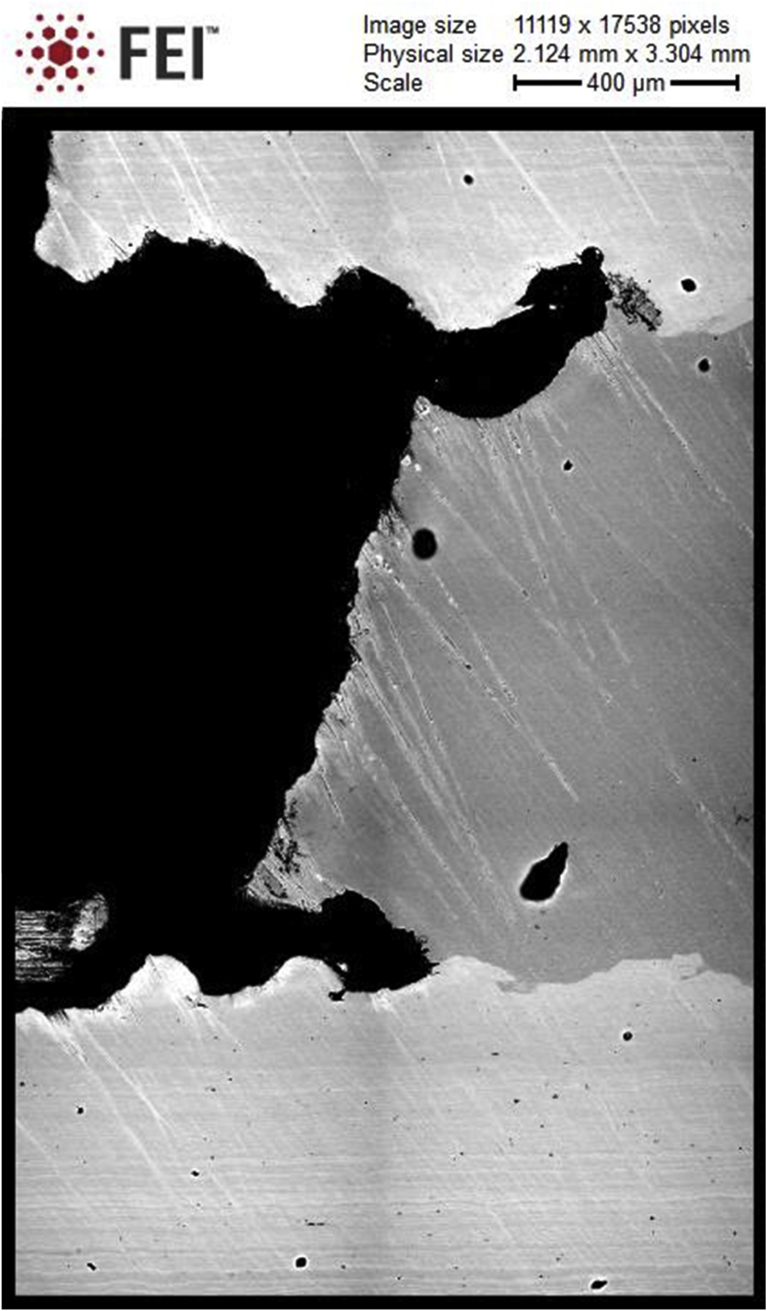
Mapped section of the sample under conditions when the corrosion reaches the third layer. 1 – external layer, 2 – internal protector, 3 – third layer, 4 and 5 – interlayer boundaries, 6 – lens.

Fig. 2 shows the longitudinal section of the deepest natural pitting with a cavity in the protector, and Fig. 3 shows the mapped section of a three-layer sample under conditions when the corrosion reaches the third layer.

**Fig. 4:**
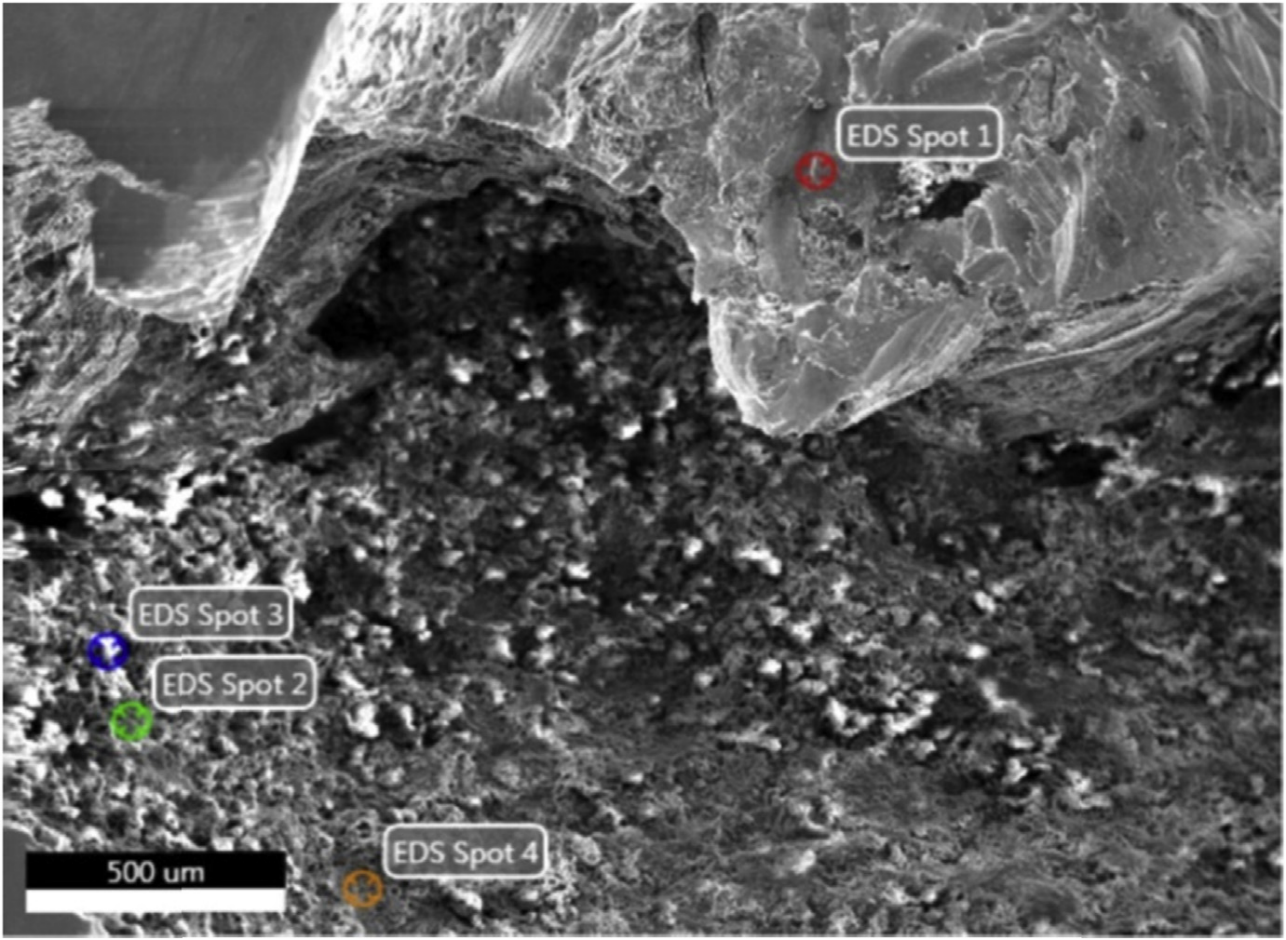
Section of the first and second layers, for which the distribution of the corrosion products' elemental composition at the spots was defined.

To study the mechanism and sequence of the corrosion damage, a micro-X-ray spectral analysis of the corrosion products was performed at particular spots of the section. Fig. 4 shows the section of the first and second layers. The elemental composition at the spots is given in Table 2.

**Table 2:** Elemental composition of the corrosion products.

Spot number	Mass fraction of an element, %											
	Fe	O	C	Cr	Ni	Ti	Si	Al	Mn	Cl	Cu	Others
Spot 1	45.56	19.77	15.94	12.03	3.56	0.87	0.7	0.63	0.58	0.36	0	0
Spot 2	43.21	45.04	7.28	0.37	0	0	0.4	0.21	0	3.3	0	0.18
Spot 3	89.47	3.68	1.78	0.35	0	0	0	0	0.53	0.2	3.98	0.01
Spot 4	64.01	26.13	7.38	0.21	0	0	0	0	0.39	0.26	0	1.63

**The electrochemical method** was used in order to determine the corrosion rate for two metals contacting in corrosive medium. The proposed method is based on a graphical analysis of the electrochemical corrosion rate and involves measurement of the corrosion current density of the contacting metals in a multilayer material with internal protector [27].

The polarization curves are plotted experimentally with the subsequent determination of the mass corrosion index of the layers' components.

**Fig. 5:**
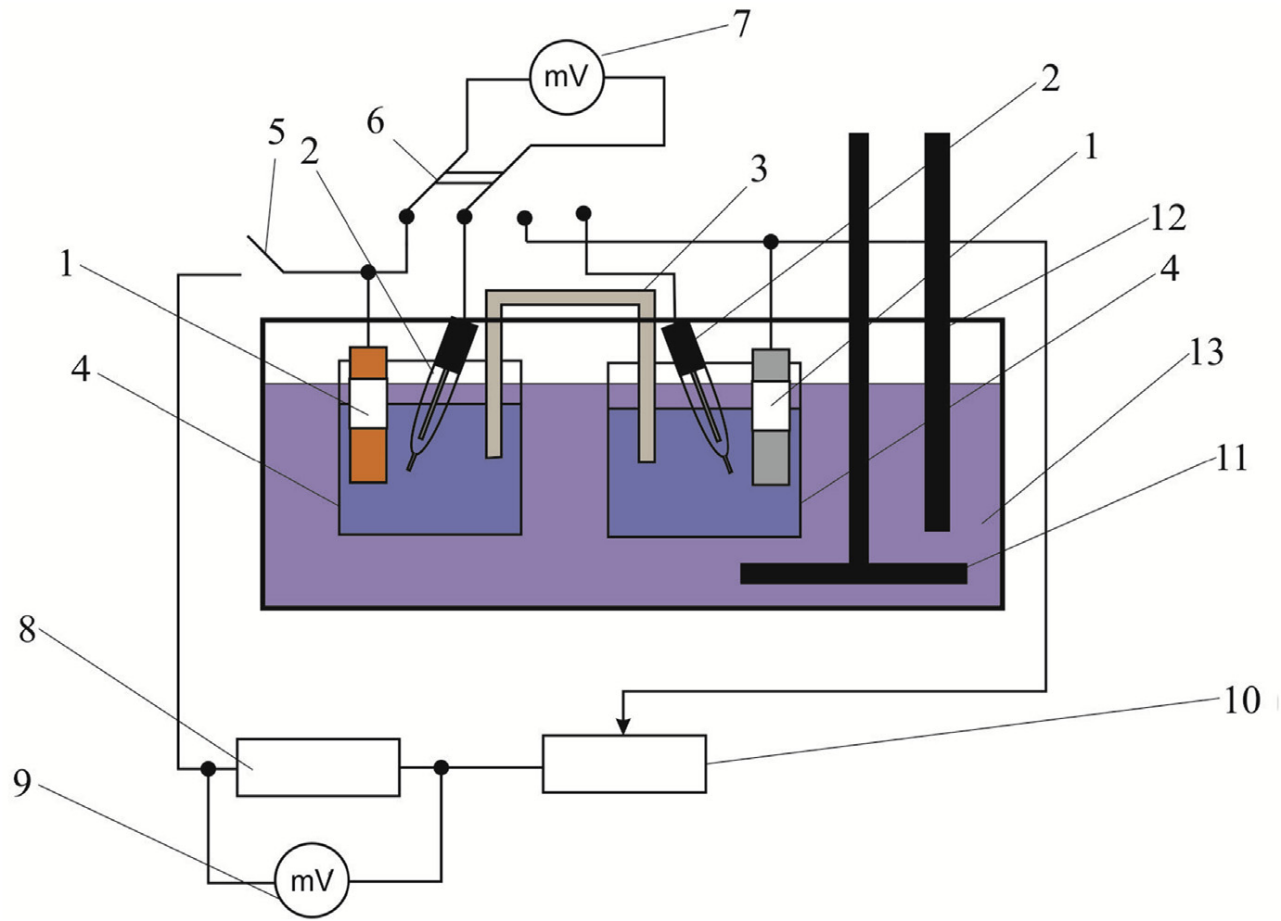
Schematic view of the assembly for electrochemical study of the corrosion elements and plotting of the corrosion diagrams: 1 – electrodes made of multilayer composite's components, 2 – reference electrodes, 3 – salt bridge, 4 – vessels filled with corrosive medium, 5 – tumbler, 6 – switch, 7, 9 – high resistance millivoltmeters, 8 – calibrated 1Ω resistor, 10 – bank of resistors, 11 – mechanical agitator, 12 – thermometer, 13 – thermostat.

The samples are placed in a temperature controlled electrochemical cell with the reference solution (Fig. 5), the electrode potentials are measured in the absence of current in the circuit and at various resistances in the range of 1–100,000 Ω. The minimum resistance is determined by the current of the corrosion element.

On the basis of the obtained electrode potentials and resistance, the corrosion current (*I*) was calculated and corrosion curve *E*_she_ = *f* (*I*) was plotted. *E*_she_ is the metal potential with regard to the standard hydrogen electrode.

The experimental procedure was performed on the metal plates with the surface area of 2.00 ± 0.05 cm^2^, made of sheet rolled initial steel 08X18H10T and steel 10. The plates were partially isolated along the length with a heat shrink tube in order to provide the required surface area of the electrode and prevent any contact of the metal surface with the “solution–air” phase interface.

With the tumbler deactivated, the electrode potentials were measured without current in the circuit (stationary potential *E*_st_), which then were recalculated with regard to the standard hydrogen electrode *E*_she_. Using the bank of resistors 10, the required electric resistance was adjusted. After closing the circuit by the tumbler *5*, the voltage drop on the calibrated resistor *8* was measured by the millivoltmeter *9*. The obtained voltage was used for calculation of the current in the circuit.

**Fig. 6:**
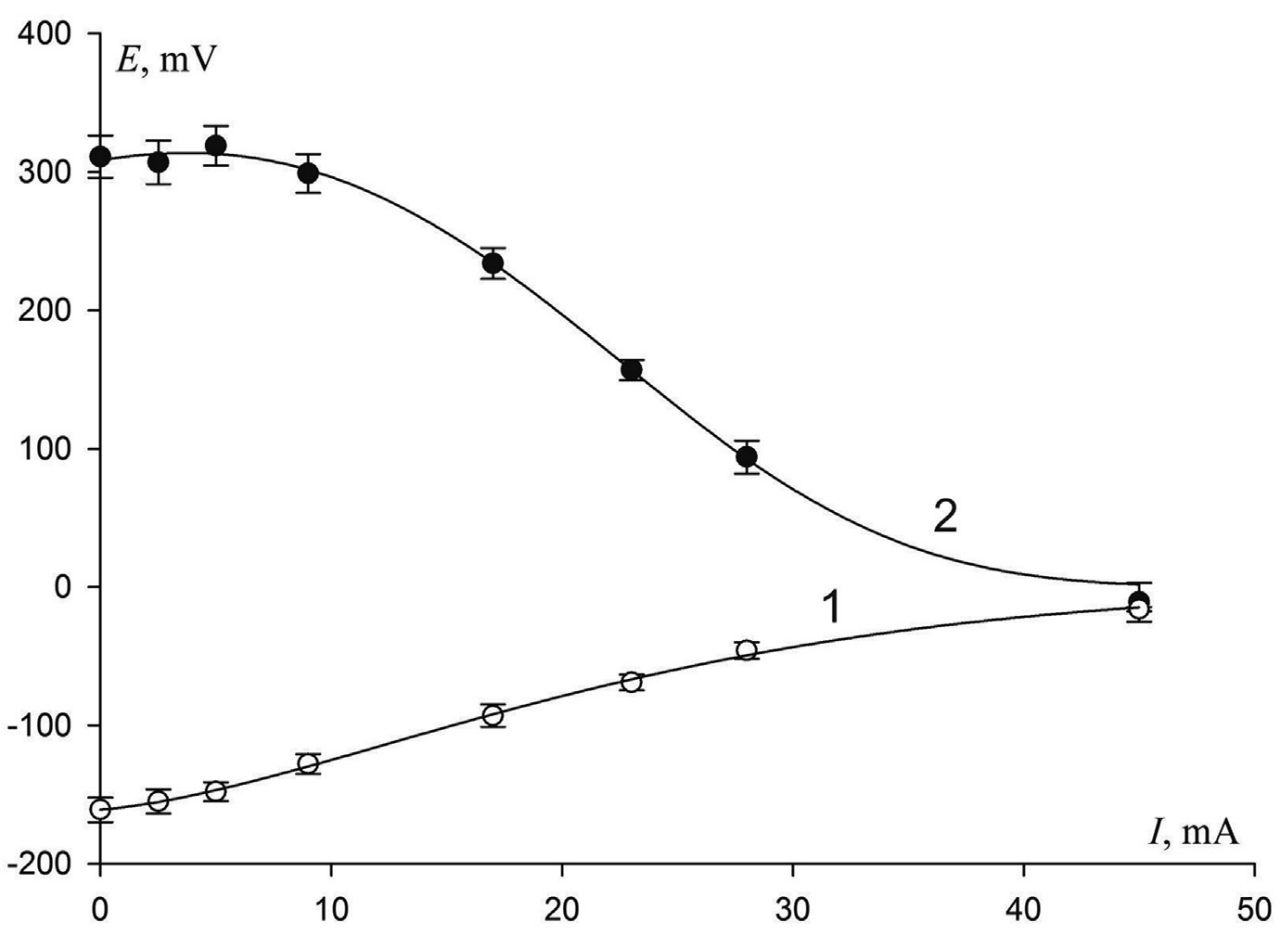
Corrosion diagram for the galvanic pair 08X18H10T (curve 2) – steel 10 (curve 1) in the iron (III) chloride solution.

Fig. 6 illustrates the corrosion diagram for the galvanic pair 08X18H10T – steel 10. As a testing medium the iron (III) chloride solution was used (density 1.049 ± 0.002 g/cm^3^).

**The chemical method** was applied to determine the corrosion rate for each layer of the multilayer material. The proposed method based on the use of the demountable type samples [28] consists in determining the mass corrosion index of each plate imitating layers of a multilayer material with internal protector.

The objects of the tests were the following:

1) demountable multilayer metallic materials with internal protector where the first and third layers were made of high alloy steel 08X18H10T, and the internal second layer – of low carbon steel 10;
2) reference samples – plates representing separate layers of the multilayer material and made of high alloy steel 08X18H10T and low carbon steel 10.

**Fig. 7:**
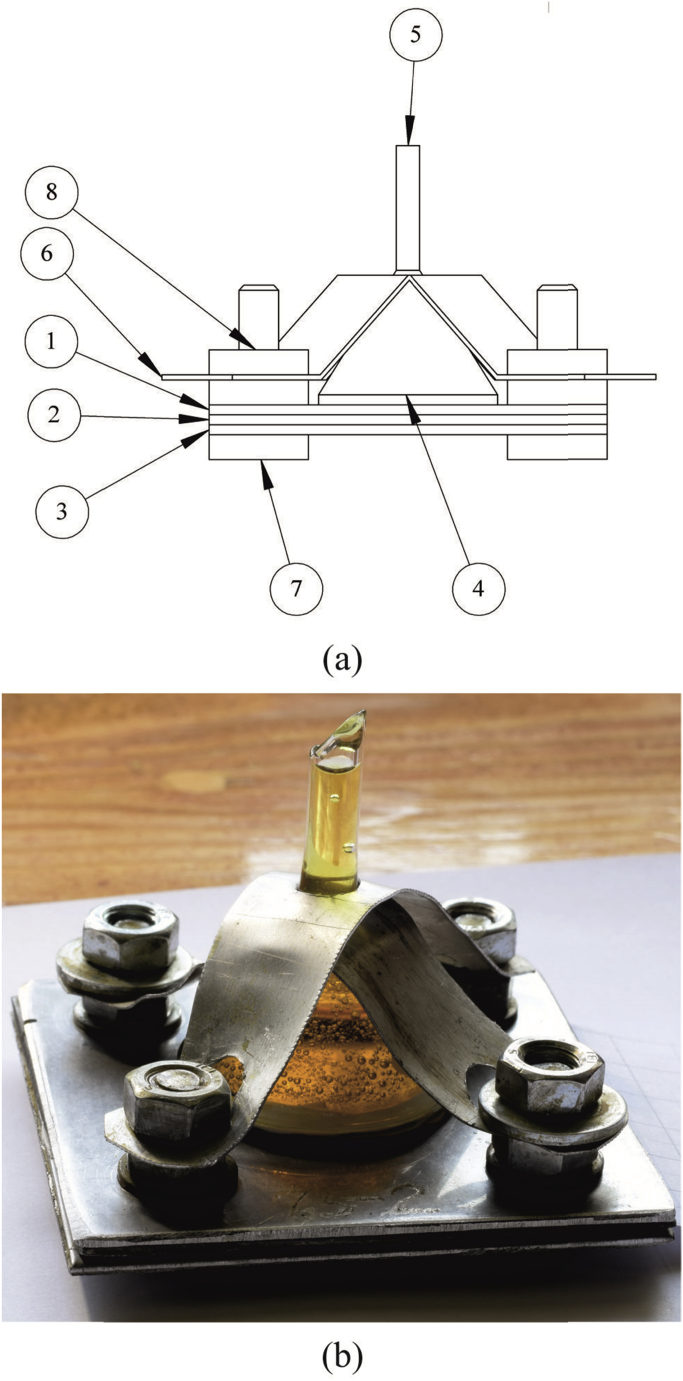
Schematic view (a) and image (b) of a sample for testing: 1 – top plate, 2 – middle plate, 3 – bottom plate, 4 – separator, 5 – glass funnel, 6 – bracket, 7 – bolt, 8 – nut.

Each demountable sample represented a bolted connection of three plates with the sizes of 80 × 80 mm and thickness from 1.5 to 2.5 mm (Fig. 7). The plates were fabricated of rolled sheets.

In the center of the upper plate made of high alloy steel a through hole was made with the diameter of 1.0 ± 0.2 mm (artificial pitting). In the center of the plate made of low carbon steel a through hole was made with the diameter *d*, in such sequence: 3.0; 5.0; 10.0; 15.0 and 20.0 mm with the accuracy of ±0.1 mm. This range of diameters, on one hand, is to simulate the corrosion development in the protector layer, shortening the testing time by modeling the various stages, and, on the other hand, to determine the beginning of pitting formation in the third layer. The conditions of corrosion attack are more stringent than the natural corrosion development, because they provide the corrosion influence on the protector by a reagent of high activity. Under natural conditions, an increase in the diameter of the lens occurs due to anodic dissolution of the metal. The number of samples should provide the possibility of statistical processing of the results.

The plates were weighed with the accuracy of 0.0001 g. The plates were combined into a pack, the gaps between the plates around the central hole were sealed. Glass funnels with the diameter of 36 mm were installed via inert separators on the high alloy steel plates of each pack above the artificial pitting. The funnel was additionally fixed with the brackets (Fig. 7) and filled with the iron (III) chloride solution.

The testing time was 720 and 2,208 h, the solution was replaced every 168 ± 2 h without detaching the samples and removing the corrosion products.

After completion of each stage the packs were disassembled, washed with water, and dried, occurrence of pitting was examined visually.

Mass corrosion indices $K_{m}^{-}$ (g/m^2^·h) were calculated on the basis of the measurements for the upper, middle and bottom plates according to the equation:

(5) $$K_{m}^{-}=\frac{\left[\left(m_{01}+m_{02}+m_{03}\right)-\left(m_{11}+m_{12}+m_{13}\right)\right]}{\tau \cdot \left(S_{1}+S_{2}+S_{3}\right)}\text{.}$$

*m*_01_, *m*_02_, *m*_03_, *m*_11_, *m*_12_, *m*_13_ are the masses of plates in three parallel samples before and after testing, respectively, (g); τ is the exposure time (h); *S*_1_, *S*_2_, *S*_3_ are the cumulative areas of the plates for three parallel samples in contact with the corrosive medium (m^2^).

The reference samples were tested under the same conditions and the same exposure time as the three-layer samples, including the replacement of the solution. After the completion of each testing stage of the reference samples, the occurrence of pitting was visually examined on the high alloy steel plates, and then they were weighed.

The mass corrosion indices of the reference samples made of low carbon and high alloy steels ($Kref_{m}^{-}$) were calculated by Eq. (5) using the mass determination data.

**Fig. 8:**
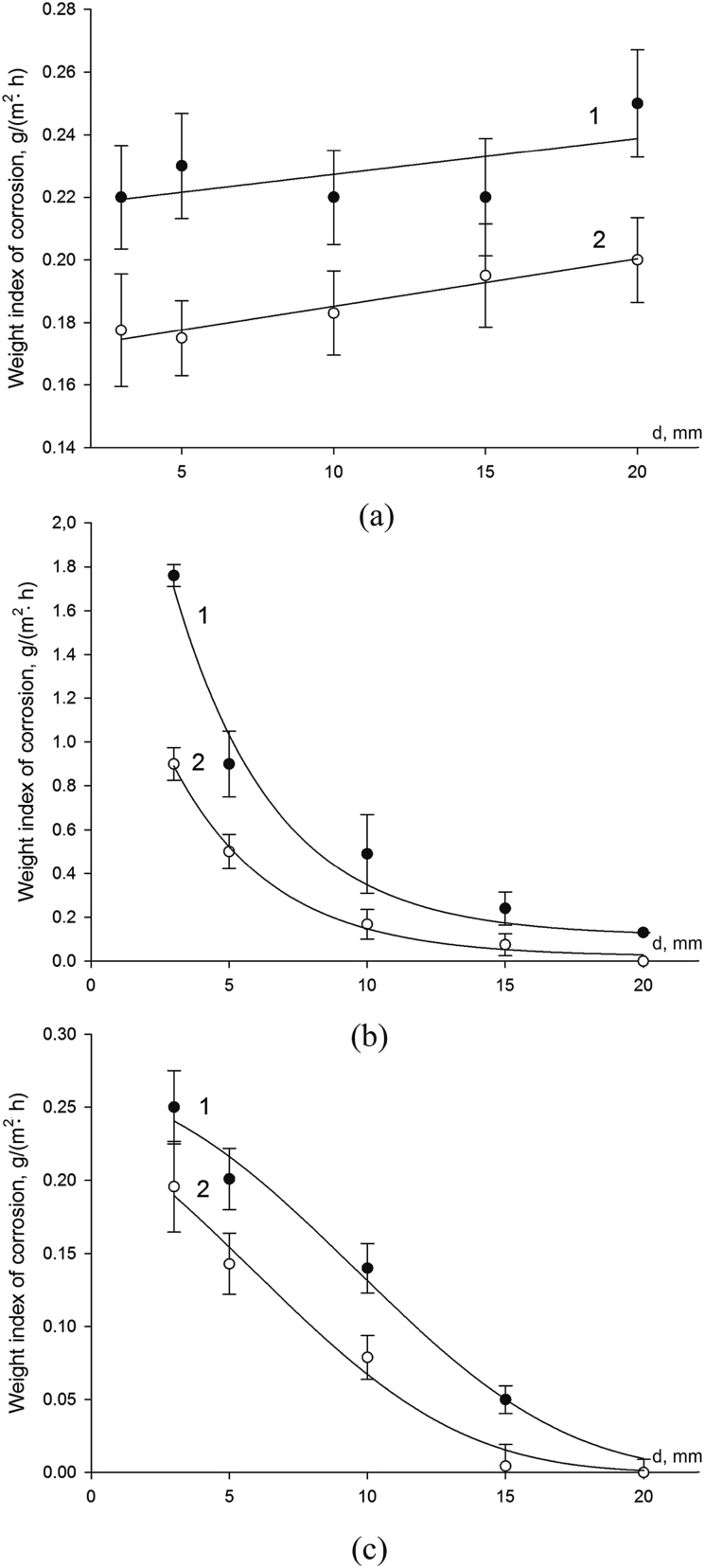
Mass corrosion index of the external (a), middle (b) and bottom (c) layer of the metallic multilayer material at different diameters (d, mm) of an artificial lens, diameter of an artificial pitting of 1 mm and exposure time of 720 h (curve 1) and 2,208 h (curve 2).

On the basis of experimental results, the mass corrosion index was plotted as a function of the lens diameter for each plate of the three-layer sample (Fig. 8).

## 4 Discussion

Electron microscope studies reveal the mechanism of corrosion processes and prove the protector's effectiveness.

According to Fig. 2, anodic dissolution in the protector formed a cavity with cross-sectional dimensions up to 5.0 mm. The dissolution boundary passed along the wave surface of the weld, at the point of contact of the first layer with the protector. The formation of the wave surface is characteristic of explosion welding. In the first layer, a pitting with a diameter of 1.5 mm was formed under the influence of aggressive medium.

This indicates that at the initial stage the corrosion damage of the outer layer is analogous to that of a monometallic material when exposed to aggressive medium. Numerous pittings are formed on the surface.

When the deepest pitting reaches the second layer, the electrochemical interaction of the two layers with the aggressive medium begins, which changes the character of the corrosive destruction. Anodic dissolution of the protector proceeds more intensively at these contact points, forming lenses up to 5 mm in diameter. When the third layer is reached, anodic dissolution of the protector develops in the transverse direction (Fig. 3).

According to Table 2, at the stage of the pitting initiation and development in the external layer and dissolution of the protector, a large number of oxo-hydroxo compounds of iron, chromium and nickel are formed, which is confirmed by the presence of oxygen at the spot 1 (Fig. 4). At the spots 2, 3 and 4, the presence of oxygen is due to the formation of poorly soluble oxygen-containing corrosion products. The presence of chlorine at these spots confirms the formation of metal hydroxochlorides. The reduction of iron content at all spots is due to its dissolution in the corrosive medium. On the pitting and lens' surfaces, the carbon content is manifold higher than the initial values of Table 1, which, apparently, is due to the lack of solubility of carbon in the test solution.

Thus, this method provides a qualitative assessment of the corrosion processes occurring in a multilayer metallic material with internal protector, and confirms the mechanism of protector's action. Nevertheless, it does not allow quantifying the corrosion rate for the layers of a multilayer material under conditions of the through damage to the external layer.

The application of the electrochemical method made it possible to plot a corrosion diagram (Fig. 6).

As it follows from the diagram, with an open circuit, the potential difference between the electrodes made of steel 08X18Н10Т and steel 10 is about 500 mV. As the external resistance decreases, the current increases and the potentials shift. The change in the potential of 08X18Н10Т steel towards the negative values is about 330 mV, and of steel 10 towards the positive values is about 150 mV. The corrosion current I_cor_ is 45 mA.

The processing of experimental data in order to calculate the degree of anodic, cathodic and ohmic control allows concluding that in the test solution the degree of cathodic control of the corrosion process is more than twice the total value of the degree of anodic and ohmic control. The cathodic process is the limiting one. The results of calculations based on the equations recommended in [23] are given in Table 3.

**Table 3:** Degrees of control of galvanic pair 08X18H10T – steel 10.

Degree of control	Value, %
Degree of cathodic control *C*_c_	68.2
Degree of anodic control *C*_a_	30.7
Degree of ohmic control *C*_R_	1.1

The developed method allows determining the absence of anodic passivation of the protector at the design stage of a multilayered material with internal protector, and substantiating the choice of layer materials experimentally.

The application of the chemical method makes it possible to determine experimentally the mass corrosion indices of each layer separately. As can be seen in Fig. 7a, the mass corrosion index of the external layer increases with the increase in the hole diameter in the protector from 0.22 to 0.25 g/(m^2^·h) at exposure in 720 h, and from 0.18 to 0.19 g/(m^2^·h) in 2,208 h. This can be attributed to the increase in the thickness of the diffusion layer. The increase in the sample's exposure time leads to the decrease in the corrosion rate of the external layer in 1.2–1.4 times, which can be stipulated by partial filling of the pitting cross section with the low soluble products resulting from the dissolution of the protector.

The mass corrosion index of the middle layer (Fig. 7b) decreases with the increase in the hole diameter from 1.76 to 0.13 g/(m^2^·h) in 720 h and from 0.90 g/(m^2^·h) almost to 0 in 2,208 h. With the increase in the exposure time, the anodic dissolution rate of the protector decreases, this is stipulated by filling of the pitting pore and artificial lens with the products of the protector's dissolution, and by the decrease in the concentration of oxidizers in the lens.

The mass corrosion index of the third layer (Fig. 7c) decreases from 0.25 to 0.003 g/(m^2^·h) at the exposure time of 720 h. The increase in the exposure time to 2,208 h leads to the decrease in $K_{m}^{-}$ from 0.19 g/(m^2^·h) to 0. This is stipulated by the variation in composition of the corrosive medium above the internal surface of the third layer.

The following conclusions can be drawn based on the analysis of this method's results:

1) Based on the external inspection of the samples, at the diameter of artificial pitting in the first layer equaling to 1 mm and the diameter of artificial lens in the protector (the second layer) from 3.0 to 20.0 mm, the dissolution of both the first and second layers was observed. On the surface of the first layer, the formation of new pitting centers occurred.

At small diameters of the lens in the second layer, the values of the mass corrosion index of the first and third layers practically does not differ, i.e. the protector equally effectively protects them from corrosion.

As the lens diameter increases, the effectiveness of the protector for the first layer decreases, which is explained by the increase in the distance from the protector's wall to the outer surface of the first layer. And the mass corrosion index of the third layer decreases significantly, which is explained by the increasing role of the diffusion limitations of the process, the difficulty in delivering the dissolved oxygen, as well as by the increase in the protector's area.

2) The corrosion rate of the external layer of the multilayer material under the considered conditions is 58–66 times lower than that of the reference samples, thus proving the protector's effectiveness.

When compared to the monometallic material, corrosion failure of the multilayer metallic material in the presence of pitting in the outer layer proceeds in different concentration-diffusion conditions. As the internal layer-protector dissolves and the size of the lens grows, the thickness of the diffusion layer (the distance from the outer surface of the external layer to the wall of the protector) increases. The latter, as well as the formation of slightly soluble products resulting from the protector's dissolution, should obviously result in the retardation of the protector's dissolution and resumption of new pitting formation on the external layer.

3) The rate of the protector's anodic dissolution decreases with an increase in the artificial lens diameter.

The developed method allows determining the relative index of corrosion resistance of a multilayer material *RI* in comparison with a monometallic material in a specific corrosive medium. It is calculated as the ratio of the time required for the first layer's through destruction and the time required for the second layer material's destruction to the lens size, at which pitting begins in the third layer, to the time required for the monometallic material's through destruction upon equal thickness:

(6) $$RI=\frac{t_{1}+t_{2}}{t_{m}}\text{.}$$

*t*_*1*_ – time required for the first layer's through destruction, *t*_*2*_ – time required for the second layer material's destruction to the lens size at which pitting starts in the third layer, *t*_*m*_ – time required for the monometallic material's through destruction.

According to the data of [15], the mass corrosion index of the material of the first layer (steel 08X18H10T) in the solution of iron (III) chloride is 14.5 g/(m^2^·h), of the second layer (steel 10) in contact with the material of the first layer – 158.7 g/(m^2^·h).

To calculate the through destruction of the first layer, the $K_{m}^{-}$ of the first layer's material in the FeCl_3_ solution was used. The calculations were carried out at a pitting diameter of 1 mm and a depth of 2 mm. At these parameters, the time *t*_*1*_ was 131.8 h. To calculate the time required for the lens growth in the second layer to a diameter of 20 mm, we used the average $K_{m}^{-}$ of the second layer's material in the solution of FeCl_3_ – 6.9 g/(m^2^·h). This value was obtained by integrating the dependence $K_{m}^{-}=f\left(d\right)$ (Fig. 7b) between d = 0 mm ($K_{m}^{-}$= 158.7 g/(m^2^·h)) and d = 20 mm. The maximum lens diameter of 20 mm is selected based on the experimental confirmation of the pitting absence in the third layer. Further growth of the lens leads to the decrease in the internal protector's effectiveness and the beginning of the pitting growth in the third layer, which agrees with the results of [12]. The obtained value of the area under the curve was used to find the average value of the mass corrosion index by plotting a rectangle of the same area with the side d = 20 mm. The results are given in Table 4.

**Table 4:** The results of the mass corrosion index calculation.

Sample exposure time, h	Lens diameter in the protector, mm	Mass corrosion index $K_{m}^{-}$, g/(m^2^·h)
720	0–20	6.88
2,208	0–20	6.87

With these parameters, the dissolution time for the internal protector to the lens diameter of 20 mm *t*_*2*_ is 5,654.0 h. The total dissolution time for the first and second layers (*t*_*1*_ +*t*_*2*_) is 5,785.8 h. Under the same conditions, the pitting growth time for the monomaterial (steel 08X18H10T) with a diameter of 1 mm and a depth of 4 mm *t*_*m*_ is 263.6 h. Thus, the relative index of corrosion resistance of the multilayer material is *RI* = 21.9.

## 5 Conclusions

The authors have proposed a set of methods for accelerated testing of a multilayer metallic material with internal protector, which allows confirming experimentally the effectiveness of the multilayer composite's architecture in general and of the protector in particular, and also predicting the material's durability under specific conditions.

Electron microscope examination of material, after exposure to corrosive environment, makes it possible to qualitatively assess the corrosion processes occurring in a multilayer metallic material with internal protector, and confirm the mechanism of the protector's action. However, this method does not make it impossible to quantitatively estimate the rate of the layers' destruction in case of the through damage to the outer layer.

The electrochemical method of corrosion tests of multilayer materials, developed by the authors, allows establishing the absence of anodic passivation of the protector at the design stage of a multilayered material with internal protector, and substantiating experimentally the choice of the layers' materials.

The chemical method based on the demountable model of a multilayer composite allows determining experimentally the values of the mass corrosion indices for each layer separately and the corrosion resistance index of a multilayer material in comparison with a monometallic material. It has been established that the relative index of corrosion resistance for the architecture of the multilayer material 08X18H10T + steel 10 + 08X18N10T was 21.9 times higher than that of the monometallic material 08X18Н10Т.

The developed methods can be used to build the architecture of a multilayer material and determine the quantitative indicators of corrosion resistance in corrosive media of various compositions.

## Declarations

### Author contribution statement

Vladimir A. Grachev, Andrey E. Rozen: Conceived and designed the experiments; Analyzed and interpreted the data; Wrote the paper.

Yuri P. Perelygin, Sergey Y. Kireev, Irina S. Los': Conceived and designed the experiments; Performed the experiments.

Andrey A. Rozen: Contributed reagents, materials, analysis tools or data.

### Funding statement

This work was supported by the Ministry of Education and Science of the Russian Federation (Project No. 10.6563.2017/8.9).

### Competing interest statement

The authors declare no conflict of interest.

### Additional information

No additional information is available for this paper.

## References

[1] A.E. Rozen, I.S. Los', Yu. P. Perelygin, L.B. Pervukhin, Yu.A. Gordopolov, G.V. Kiriy, P.I. Abramov, S.G. Usatyi, D.B. Kryukov, O.L. Pervukhina, I.V. Denisov, A.A. Rozen, Multilayer material with enhanced corrosion resistance (variants) and methods for preparing same. Eurasian Patent 016878, C23F 13/00, B 32B 7/02, issued June 30, 2012.

[2] A.E. Rozen, I.S. Los', Yu. P. Perelygin, L.B. Pervukhin, Yu.A. Gordopolov, G.V. Kiriy, P.I. Abramov, S.G. Usatyi, D.B. Kryukov, O.L. Pervukhina, I.V. Denisov, A.A. Rozen, Multilayer material with enhanced corrosion resistance (variants) and methods for preparing same. Patent 10-1300674 KIPO, Reg. date: August 21, 2013.

[3] Russian Standard GOST 9.912–89 (1990). Unified System of Corrosion and Ageing protection. Corrosion Resistant Steels and Alloys. Methods of Accelerated Tests for Resistance to Pitting Corrosion.

[4] ASTM G48-11 (1995).

[5] BS EN ISO 11463:2008 (2008). Corrosion of Metals and Alloys. Evaluation of Pitting Corrosion.

[6] C.E. Schell III, A. Weisstuch, Method and Apparatus for Measuring Pitting Corrosion Tendencies. US Patent 3878064, Int. Cl. G0lN27/46, Pa, Publication date: April 15, 1975.

[7] G.A. Martinchek, M.R. Yaffe, Detection of Pitting Corrosion. US Patent 6015484, Int. Cl. G01N17/02, Pa, Publication date: January 18, 2000.

[8] Y.Y. Ushakova, N.M. Tutukana, I.K. Marshakov, Pitting Corrosion Test Method. RU Patent 1718048, Int. Cl. G01N17/00, Publication date: March 7, 1992.

[9] V.V. Ryzhakov, V.A. Kupryashin, O.Y. Baikov, Method for predicting the durability of products made of chromium-nickel stainless steels in halogen-containing media. RU Patent 2403557, Int. Cl. G01N17/00, Publication date: January 10, 2010.

[10] L.M. Eswara, L.R. Boregowda, T. Ekambaram, System and method for prediction of pitting corrosion growth. US Patent 7609874, Int. Cl.4 G06K9/00, Publication date: October 27, 2009.

[11] Li H., Garvan M.R., Li J., Echauz J., Brown D., Vachtsevanos G.J. (2014). Imaging and information processing of pitting-corroded aluminum alloy panels with surface metrology methods. Annual Conference of the Prognostics and Health Management Society.

[12] Los' I.S. (2016). Otsenka korrozionnoi stoikosti mnogosloinykh metallicheskikh materialov. Voprosy materialovedeniya.

[13] Grachev V.A., Rozen A.E., Perelygin Y.P., Rozen A.A. (2017). Multilayer metallic material with specific properties and the technology of its production. Russ. Metall..

[14] Grachev V.A., Rozen A.Y., Kozlov G.V., Rozen A.A. (2016). Mechanism of pitting corrosion protection of metals and alloys. Orient. J. Chem..

[15] Los' I.S., Perelygin Yu. P., Rozen A.E., Kireev S. Yu. (2015). Mnogosloinye Korrozionno-stoikie Materialy.

[16] Perelygin Yu. P., Rosen A.E., Los' I.S., Kireev S. Yu. (2014). A new corrosion-resistant multilayer material. Prot. Met. Phys. Chem. Surf..

[17] Rozenfeld I.L. (1970). Korroziya I Zashchita Metallov.

[18] Tomashov N.D., Chernova G.P. (1986). Teoriya Korrozii I Korrozionnostoikie Konstruktsionnye Splavy.

[19] Scorceletti V.V. (1973). Teoreticheskie Osnovy Korrozii Metallov.

[20] Tomashov N.D., Zhuk N.P., Titov V.A., Vedeneeva M.A. (1961). Laboratornye Raboty Po Korrozii I Zashchite Metallov.

[21] Russian Standard GOST 5632–2014 (2015). Stainless Steels and Corrosion Resisting, Heat-resisting and Creep Resisting Alloys.

[22] Russian Standard GOST 1050–2013 (2014). Metal Products from Nonalloyed Structural Quality and Special Steels.

[23] Kershenbaum V.S. (1992).

[24] ASTM A 276–06 (2006). Standard Specification for Stainless Steel Bars and Shapes.

[25] ASTM A 29/A 29M–05 (2005). Standard Specification for Steel Bars, Carbon and Alloy, Hot-Wrought, General Requirements.

[26] Lysak V.I., Kuzmin S.V. (2005). Svarka Vzryvom.

[27] Y.P. Perelygin, S.Yu. Kireev, I.S. Los', A.E. Rozen, M.Y. Panin, Device for electrochemical study of metal corrosion. RU Patent 2533344, Int. Cl. G01N17/02, Bull. No. 32, 2014.

[28] Kireev S. Yu., Los' I.S., Rozen A.E., Perelygin Yu. P. (2017). Metodika korrozionnykh ispytanii mnogosloinogo metallicheskogo materiala. Korroziya Materialy Zashchita.

